# Retraction: SiC ultra -thin films for high-performance quantum spacecraft applications fabricated via nanosecond pulsed laser deposition and doping

**DOI:** 10.1371/journal.pone.0351888

**Published:** 2026-06-22

**Authors:** The PLOS One Editors

The *PLOS One* Editors retract this article [1] due to concerns about:

- Citation of numerous unretrievable references, including References 7, 8, 9, 10, and 11.
- Numerous references are repeated in the References list, for example the article cited as Reference 3 is incorrectly duplicated as References 18, 23, 27, 30, and 32.
- Compliance with PLOS policies on Authorship and Artificial Intelligence Tools and Technologies.

We regret that the issues were not identified prior to the article’s publication.

The second author declared that the citation errors arose due to issues pertaining to journal access, manuscript formatting using referencing software, and intelligence tools used to compile and format the reference list. The authors provided a revised reference list in their response; however, the editors consider the errors to be too extensive to resolve with a correction.

PKK, PKHG, VVW, AK, and DC did not agree with the retraction. SG and TNK either did not respond directly or could not be reached.
